# CRISPR dynamics during the interaction between bacteria and phage in the first year of life

**DOI:** 10.1099/mgen.0.001053

**Published:** 2023-07-04

**Authors:** Jiqiu Wu, Hanyun Zhang, Rui Gan, Yan Xia, Fan Zhang, Daoming Wang, Jingyuan Fu, Timothy G. Barraclough

**Affiliations:** ^1^​ Department of Life Sciences, Imperial College London, Silwood Park Campus, Ascot, Berkshire SL5 7PY, UK; ^2^​ West China Biomedical Big Data Center, West China Hospital/West China School of Medicine, Sichuan University, Chengdu 610041, PR China; ^3^​ Department of Genetics, University Medical Center Groningen, University of Groningen, Groningen 9713 AV, Netherlands; ^4^​ Division of Molecular Pathology, The Institute of Cancer Research, London SM2 5NG, UK; ^5^​ Changping Laboratory, Beijing 102206, PR China; ^6^​ 01Life Institute, Shenzhen 518000, PR China; ^7^​ HIT Center for Life Sciences, School of Life Science and Technology, Harbin Institute of Technology, Harbin 150001, PR China; ^8^​ Department of Pediatrics, University of Groningen, University Medical Center Groningen, Groningen 9713AV, Netherlands; ^9^​ Department of Biology, University of Oxford, 11a Mansfield Rd, Oxford OX1 3SZ, UK

**Keywords:** CRISPR, gut microbiome, Bacteria, Metagenomics, spacers, Evolution

## Abstract

Gut microbiomes in infancy have a profound impact on health in adulthood. CRISPRs play an essential role in the interaction between bacteria and phages. However, the dynamics of CRISPRs in gut microbiomes during early life are poorly understood. In this study, using shotgun metagenomic sequencing data from 82 Swedish infants’ gut microbiomes, 1882 candidate CRISPRs were identified, and their dynamics were analysed. We found large-scale turnover of CRISPRs and their spacers during the first year of life. As well as changes in relative abundance of the bacteria containing CRISPR, acquisition, loss and mutation of spacers were observed within the same CRISPR array sampled over time. Accordingly, the inferred interaction network of bacteria and phage was distinct at different times. This research underpins CRISPR dynamics and their potential role in the interaction between bacteria and phage in early life.

## Data Summary

Metagenomic sequencing reads data used in this research were downloaded from GigaDB (http://gigadb.org/dataset/100145, downloaded over 20–27 May 2019) generated by an infant gut microbiome study [1]. All code is available at https://github.com/JiqiuWu/CRISPR_2022.

**Impact Statement**

Clustered regularly interspaced short palindromic repeats (CRISPRs) provide an immune system for bacteria to defend themselves against viruses. The CRISPR array contains spacers that encode RNAs matching to the sequence of invading viruses. These sequences direct cleavage of viral sequences by CRISPR-associated (Cas) proteins. As a result, changes in CRISPR arrays could play an important role shaping interactions between bacteria and phage. This study explores the potential role of CRISPRs in large-scale changes in the infant gut microbiome during the first year of life. Previous work observed that bacteria and phage composition is highly dynamic during the first few months of life. Our results show that CRISPR arrays are also dynamic during this period. We observed gains, losses and mutations in CRISPR spacer regions that reflect bacterial evolution in response to changing phage exposure. This work extends understanding of the mechanisms of bacteria-phage arms races in the gut, and useful baseline data for planned uses of phage therapy to manipulate gut microbiomes.

## Introduction

Clustered regularly interspaced short palindromic repeats (CRISPRs) [2] are an important adaptive immune system found in most archaea and many bacteria for resistance against phages [3]. Generally, the CRISPR array itself provides genetic memory of infection, coupled to Cas (CRISPR-associated) proteins that provide the catalytic core of the system. The CRISPR array is composed of repetitive sequences (repeats) separated by variable sequences (spacers). The spacer sequences are derived from invading mobile genetic elements, such as bacteriophages [4]. The crRNAs encoded by the spacers guide the complexes of Cas proteins to the complementary bacteriophage target sequences that match the spacers. Cleavage of the sequences is then catalysed by various Cas enzymes [5]. During the arms race between bacteria and phages, phages can overcome CRISPR-Cas-mediated resistance by a simple point mutation in the target sequence [6], which leads hosts to incorporate more spacers in response to increasing phage mutation accumulation [7]. Bacteria-phage dynamics as mediated by CRISPRs could therefore play an important role in shaping the dynamics and functioning of bacterial communities.

One such case comprises the dynamics of the gut microbiome in infancy, which have a profound impact on health in adulthood [8]. As infants grow, their diet changes. After weaning they are exposed to a more complex diet [1, 9] and their gut microbiome shifts to become more diverse and stable. At the same time, a highly dynamic interaction between bacteria and phage begins from birth in a high predator–low prey manner [10]. Between 1 and 2 weeks of age, the relative abundance of different viral sequences changes dramatically [11]. Later on, there is a slight decrease in double stranded DNA (dsDNA) phage diversity, specifically of *Siphoviridae* phages, and an increase in single stranded DNA (ssDNA) phage diversity [12, 13]. Knowledge on gut phages and interaction between bacteria-phages is quite limited, however, especially the role of CRISPR arrays [14].

During the interactions between bacteria and phage, CRISPR arrays can change their spacers rapidly [15]. There have been some studies on CRISPRs of single bacterial species or genera experimentally, such as S *treptococcus thermophilus* [16], *Streptococcus* [17], and *Bifidobacterium longum* [18], which show that CRISPRs enhance phage resistance by spacer expansion and loss. Recently, several studies have used metagenomics data to investigate the enlarged CRISPR composition [19] and to infer the network of bacteria-phage interactions [20] in the gut microbiome community. However, the dynamics of CRISPR in the gut microbiome of infants over time is largely unknown.

In this study, we used shotgun metagenomic sequencing data to survey the dynamics of CRISPR in the whole gut microbiome community of 82 Swedish infants from birth to 12 months old [1]. We analysed how the number and composition of CRISPR and spacers changed over time *in silico*. Our results showed that CRISPRs were widely distributed in the dominant bacteria in the gut and the number of spacers and CRISPRs increased with the increased richness of the bacterial community as infants grew up. Interestingly, the composition of CRISPRs and spacers fluctuated over time. More specifically, both expansion and loss of spacers in CRISPRs were observed. Further, we found cases of a single nucleotide mutation between spacers in one CRISPR array. As a result, the inferred network of bacteria-phage interaction also changed greatly during the first year of life. Taken together, this work enhances the understanding of the CRISPR dynamics of human gut microbiome in early life.

## Methods

### Metagenomic dataset

All the metagenomic sequencing reads data used in this research were downloaded from GigaDB (http://gigadb.org/dataset/100145, downloaded over 20–27 May 2019) generated by an infant gut microbiome study [1]. The authors extracted the DNA, constructed the library, and shotgun-sequenced stool samples 98 infants sampled longitudinally during the first days of life and at 4 and 12 months of age [1]. For our study, some subjects with incomplete metadata were filtered out, such as those missing antibiotic usage and feeding pattern. In total, metagenomic data from 82 infants at birth, 4 months and 12 months were included (see metadata in Additional file one, available in the online version of this article in Supplementary Information).

### *De novo* assembly of contigs

Adaptor contamination, low-quality reads, and host contaminating reads were removed from the raw sequencing reads by Bäckhed and colleagues [1]. We reassembled the clean reads *de novo* using MEGAHIT [21] with the option *–no-mercy*, which recovers the sequences at very low depth and strengthens the contiguity of low-depth regions for metagenomics assembly. It is important here to recover a taxonomically representative sample, as many bacteria will be present at low frequency. The community size was defined as the sum of the base-pairs in all assembled contigs based on the MEGAHIT output and it described the total number of the detected nucleotides in the community.

### Bacterial metagenomics analysis

At the whole-metagenome level, we characterized taxonomic profiles by MetaPhlAn2 [22] with the default parameters, which searches for unique clade-specific marker genes. The resulting output from MetaPhlAn2 provided us with the relative abundance of each species present in each sample. To investigate changes in bacterial composition over time, bacterial richness (α-diversity) was calculated and principal coordinates analysis (PcoA) was performed on Bray–Curtis dissimilarities as a measure of β-diversity using the R package *vegan* [23]. To judge whether sequencing depth was adequate to infer composition, the *ape* package [24] was used to conduct rarefaction curves with 500 permutations.

### Detection of CRISPR arrays

CRISPR arrays were detected by CRISPRCasFinder [25] based on ‘repeat – spacer’ like structure with the default parameters. CRISPRCasFinder can detect the shortest CRISPR structures, but the background of spurious candidates can be very high [26]. CRISPRCasFinder includes a rating system based on several criteria to discriminate spurious CRISPR-like elements from CRISPR candidates. CRISPR arrays with evidence-levels 3 and 4 were considered as highly likely candidates, whereas evidence-levels 1 and 2 indicate potentially invalid CRISPR arrays. In addition, recent evidence suggests that isolated CRISPRs that lack Cas genes in their vicinity region can be non-functional (orphan), or work with a distant Cas locus in the same genome [27], which is hard to identify with shotgun sequencing data. Therefore, we restricted analysis to CRISPRs with evidence level ≥3 and containing at least one Cas protein. The density of CRISPRs was defined as the number of CRISPRs divided by the community size after assembly and it described how many of all nucleotide bases in a community are CRISPRs. The output of CRISPRCasFinder included CRISPR array sequence, spacer sequence, Cas Type, Cas proteins and so forth. However, for the consistency of Cas Type, we manually corrected it to define Cas proteins according to the latest classification system [28].

### Taxonomic assignments of bacterial genome and phage target of each CRISPR

Contigs with CRISPRs were queried against the NCBI NT database using BLASTn [29] (*e*-value cutoff: 1E−5, word size cut off: 100, and identity percentage: 70) to determine the taxonomy of cCRISPRs-containing bacteria. Spacers were queried against the EarlyVir (https://copsac.com/earlyvir/f1y/all.curated.vOTUs.earlyvir.fna) database [30], using BLASTn for short task (*e*-value cutoff: 1E-5, bit scores: 50, which roughly correlates to two-nt differences over the 30-nt average length of the spacers [17]) in order to identify the phages targeted by the spacers. The Sankey plot of top 20 genus was visualized by the R package *alluvial* [31]*,* and the width indicated the relative abundance.

### CRISPR array analysis and spacer analysis

Information on the CRISPR arrays and spacers was extracted from CRISPRCasFinder output json files using in-house Python script. The number of cCRISPRs and spacers in the gut microbiome of each infant at different times was summarized. To assess if differences between treatment groups were significant, data were analysed using one-way analysis of variance (ANOVA) followed by Tukey’s post-hoc multiple comparison tests in R. Results were considered significant as follows: *P<0.05, **P<0.01, ***P<0.001. The association between the number of CRISPRs and spacers were evaluated using the R’s lm function. The Jaccard index of the overlap of spacers and bacterial species between different time points were calculated using in-house function in R. The plots were visualized using ggplot2 package in R [32].

### Longitudinal analyses of the putative identical CRISPR array

In order to investigate the dynamics of spacers and CRISPRs in the identical CRISPR array, putative identical CRISPR arrays at multiple time points were selected based on the following criteria: they are detected in (1) the same infant but at different time points and in (2) the same bacterial genome, (3) their Cas protein structure is the same, (4) the *e*-value of BLASTn is less than 1E−5 and (5) with complete upstream and downstream flank sequences. The CRISPR arrays were reconstructed manually and three groups of CRISPRs meeting these criteria were highlighted. Single nucleotide dynamics of the spacers in putative identical CRISPR arrays were analysed by comparing the similarity of the unique spacers using BLASTn.

### Interaction analysis between bacteria and bacteriophages

To infer the interaction between known bacteria and known bacteriophages, the CRISPRs with spacers whose bacteriophage targets were known were used to detect the bacterial host. The network and centrality were inferred and visualized using *igraph* package [33] in R. The network graph was organized using the function layout_with_kk() and the Kamada-Kawai layout algorithm.

## Results

### CRISPRs were widely distributed in the predominant bacteria in the gut

This study included 82 infants’ clean metagenomic sequences at birth, 4 months, and 12 months (the workflow summary is in Fig. S1a, metadata of all the infants is in Additional file 1). Reads were assembled into 6534.976±5104.108 contigs, 13377.88±7217.095 contigs, and 32904.17±13 349.03 contigs per sample at birth, at 4 months, and at 12 months, respectively. The average N50 is 50439.48±48395.57, 27741.4±17 054.82, and 16045.74±8776.578 per sample at birth, at 4 months, and at 12 months, respectively (the quality of assemblies is in Additional file 2 and Fig. S1b). From these assemblies, 1882 candidate CRISPRs (cCRISPRs) were identified (sequences in Additional file 3).

**Fig. 1.**
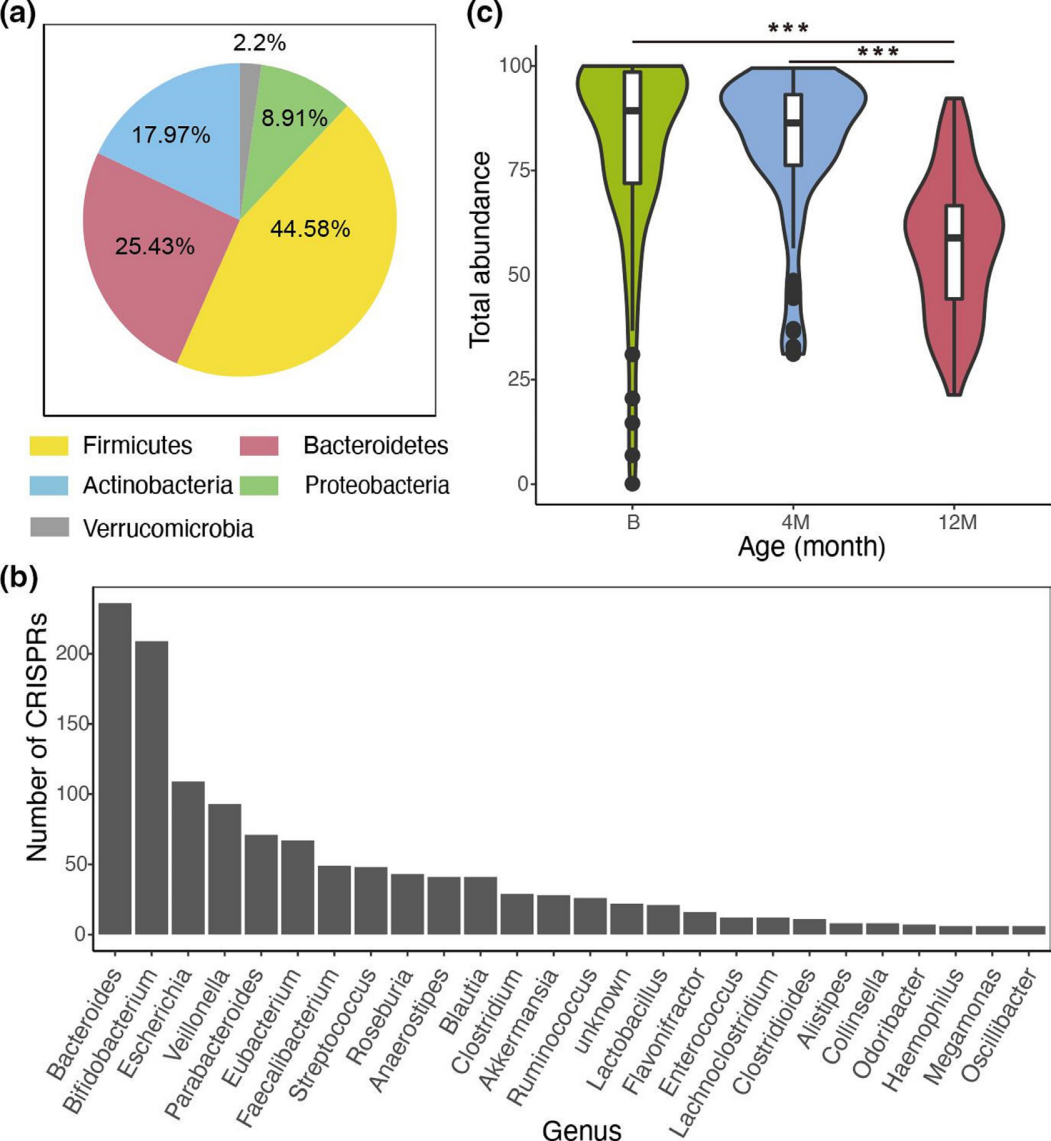
cCRISPRs were widely distributed in the dominant bacteria in the gut. (**a**) The phyla that contained cCRISPRs. Firmicutes and Bacteroidetes contained the most cCRISPRs. (**b**) The genera that contained cCRISPRs. *Bacteroides* and *Bifidobacterium* contained the most cCRISPRs. (**c**) The total relative abundance of cCRISPRs-containing bacterial species. ANOVA test: Df=2, F value=40.18, *P* value<0.0001 . Tukey test: 4 M-12M: *P* value=0.00; B-12M: *P* value=0.00.

The bacteria hosts (cCRISPRs-containing bacteria) could be assigned for most of the cCRISPRs (1274/1882, 67.69 %, host information is in Additional file 4). cCRISPRs were widely distributed in the top five predominant phyla, including Firmicutes, Bacteroidetes, Actinobacteria, Proteobacteria, and Verrucomicrobia, (Fig. 1a), and 48 prevalent genera (Fig. 1b). At phylum level, Firmicutes (568, 44.58 %) and Bacteroidetes (324, 25.43 %) contained the highest number of cCRISPRs. However, no CRISPR array was found in four phyla Euryarchaeota, Deinococcus-Thermus, and Fusobacteria. At genus level, the top five genera were *Bacteroides* (236, 18.52 %)*, Bifidobacterium* (209, 16.41 %)*, Escherichia* (109, 8.56 %)*, Veillonella* (93, 7.30 %)*,* and *Parabacteroides* (71, 5.57 %). In total, 63 species of cCRISPRs-containing bacteria together accounted for an average of 80.37±23.09, 80.69±18.03, and 57.00±16.50 relative abundance of the total microbial composition at birth, 4 months, and 12 months, respectively (Fig. 1c). Accordingly, CRISPRs were widely distributed in the dominant bacterial taxa.

### The number of CRISPRs and spacers increased with bacterial richness

**Fig. 2.**
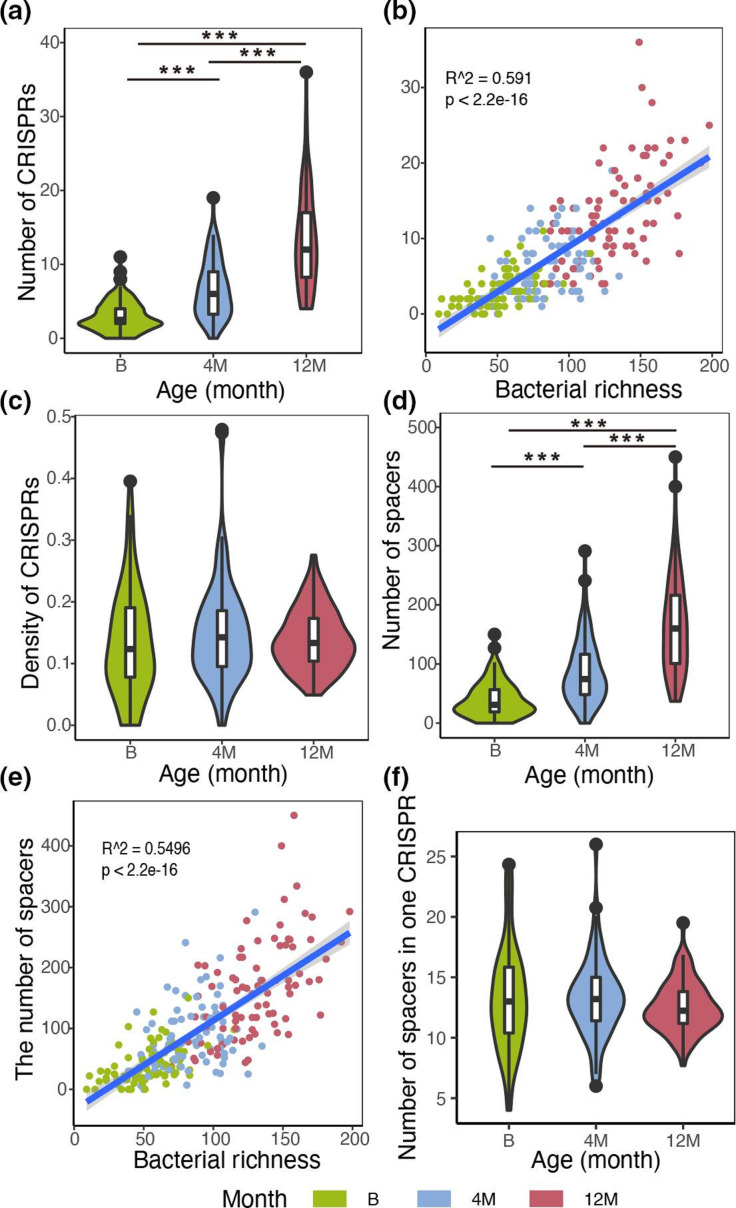
The number of CRISPRs and spacers increased with bacterial richness. (**a**) The number of cCRISPRs increased with age. ANOVA: Df=2, F value=104.3, *P* value<2e-16; (**b**) Strong association between the number of cCRISPRs and bacterial richness (R^2^=0.591, *P*<2.2e-16). (**c**) The density of CRISPRs remained constant. (**d**) The number of spacers increased with age. ANOVA: Df=2, F value=90.59, *P* value<2e-16; (**e**) Strong association between the number of spacers and bacterial richness (R^2^=0.5496, *P*<2.2e-16). (**f**) The number of spacers in one CRISPR remained constant.

Emerging evidence has shown that the diversity of infant gut microbiome increases over time. Our results were in line with the previous findings. An increase in both α-diversity (rarefaction curve of bacterial richness was shown in Fig. S2a) and community size (defined in Methods, Fig. S2b) with age was observed. The number of distinct cCRISPRs also increased with age (Fig. 2a) as a result of its strong association with bacterial richness (Fig. 2b), and as would be expected: more bacterial taxa equates to more cCRISPRs. However, the density of CRISPRs (defined in Methods) over time showed no such trend (Fig. 2c) and remained constant.

The spacers in CRISPR array encode crRNAs that guide the complexes of Cas proteins to cleave or degrade the genetic material complementary to the spacer. The number and composition of spacers determines the ability to resist foreign invasion. Next, we also studied how the number of spacers in cCRISPRs changed over time. In total, 23 894 spacers were identified and 3264 of them were found at birth, 7157 at 4 months, and 13 473 at 12 months (Fig. 2d), respectively. Among them, 17 269 spacers were unique (sequences of these spacers in Additional file 5), and the length of spacers mostly distributed from 30 bp to 35 bp. Association analysis demonstrated that the number of spacers also increased as bacterial richness increased (Fig. 2e), in line with the increase in the number of cCRISPRs. However, the number of spacers in one cCRISPR showed no trend over time and was stable (Fig. 2f).

### Turnover in the composition of cCRISPRs and spaces over time

Although it is to be expected that the total number of cCRISPRs and spacers should scale directly with overall bacterial richness, there are still several ways that numbers of cCRISPRs and spacers could change over time: 1) increased relative abundance of cCRISPRs-containing bacteria could cause the number of CRISPRs and spacers to increase with them, 2) colonization of new bacteria could bring in new CRISPRs and spacers, and 3) the number of either cCRISPRs or spacers could change within a single bacterial taxon over time. Therefore, we performed further analyses to explore these alternatives.

**Fig. 3.**
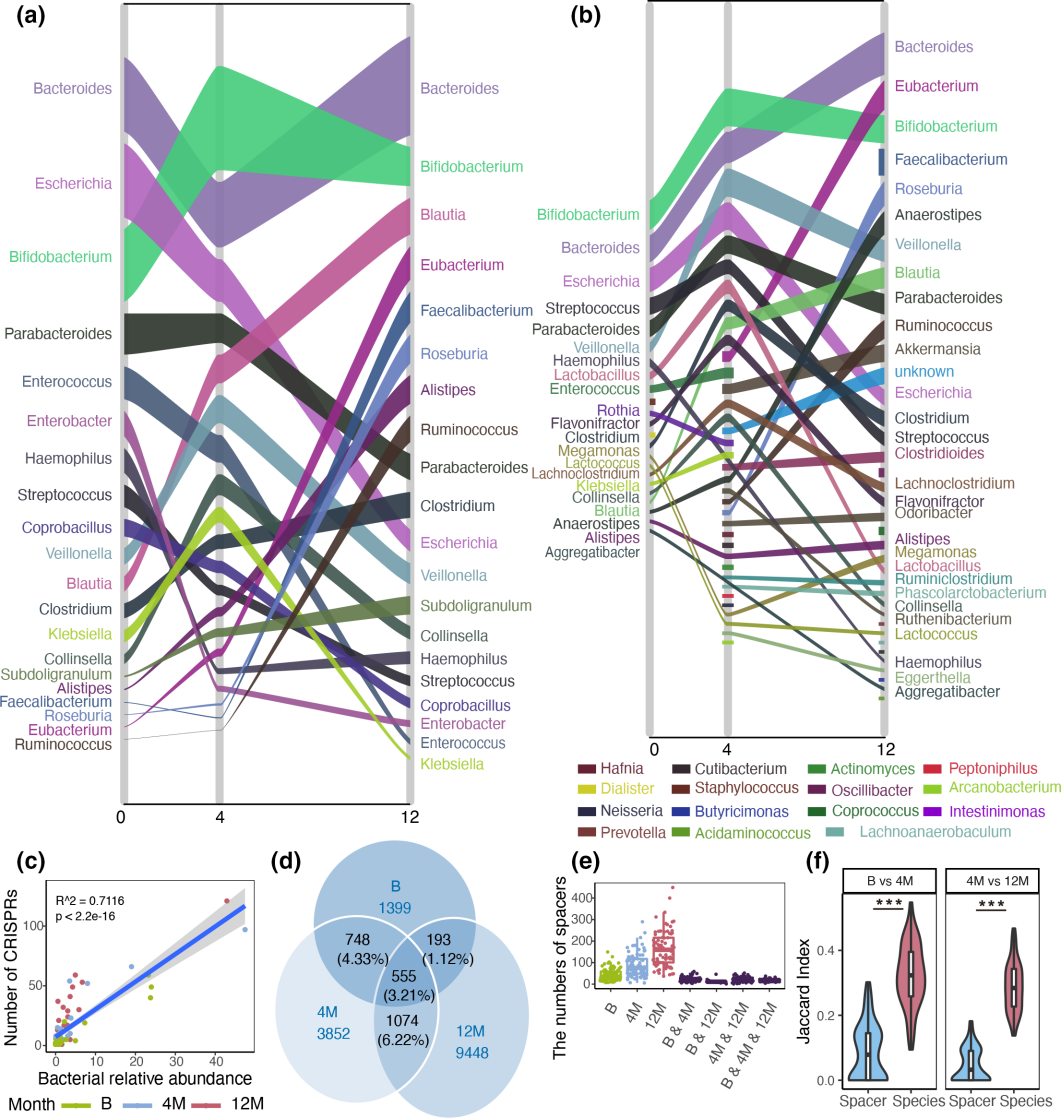
The composition of cCRISPRs and spacers changed greatly over time. (**a**) Sankey plot showing changes in bacterial relative abundance. (**b**) Changes in the number of cCRISPRs in each bacterial genus. (**c**) Strong association between the number of CRISPRs in one bacteria genus and corresponding bacterial relative abundance (Adjusted R^2^=0.7116, *P*=2.2e-16). (**d**) The Venn diagram displayed the number of shared and unique spacers at different time points among all the samples. (**e**) The boxplot depicts the number of shared and unique spacers at different time points at individual level. Green colour, blue colour and red indicate unique spacers at birth, 4 months and 12 months, respectively. Purple colour indicates the shared spacers between different time points. (**f**) The Jaccard index of spacers and species between at birth and 4 months and between 4 months and 12 months individually. The range of Jaccard index is 0 to 1. 0 means no similarity, 1 means no difference. Two-tailed unpaired Mann-Whitney U test between at birth and 4 months: W=258.5, *P*<2.2e-16; between 4 months and 12 months: W=12.5, *P*<2.2e-16.

First, over time, we found a concordant increase in relative abundance and the number of cCRISPRs in various bacteria genera, such as, *Bifidobacterium, Blautia, Parabacteroides*, *Ruminococcus,* and *Veillonella* (Fig. 3a, b, relative abundance and the number of cCRISPRs in various bacteria genera were shown in Additional file 6 and Additional file 7). Other genera such as *Bacteroides* and *Escherichia* showed no correspondence. Association analysis confirmed the strong correlation between the relative abundance of cCRISPRs-containing bacteria and the number of their containing cCRISPRs (Fig. 3c). Second, as new bacterial colonised, new cCRISPRs were brought in with new genera, such as *Eubacterium, Faecalibacterium,* and *Roseburia *. At birth, their overall relative abundance in all samples were almost zero. Later at 4 months, their abundance grew a little, and increased larger at 12 months (Fig. 3a). Corresponding to this, the number of cCRISPRs in these bacteria increased from 0 at birth (Fig. 3b).

Both these findings are in line with simple scaling with the relative abundance of the bacterial taxa. Nevertheless, we found several bacteria that initially contained cCRISPRs but lost them later (Fig. 3b). For example, *Staphylococcus* and *Dialister* contained cCRISPRs only at birth. Many bacteria only contained cCRISPRs at 4 months, such as, *Peptoniphilus, Neisseria, Hafnia, Arcanobacterium,* and *Actinomyces *. This indicated species with cCRISPRs probably disappeared in microbial community over time.

Next, we investigated changes in spacers in all samples and individually. We found, among the 17 269 unique spacers across the whole dataset, only 3.21 % (555/17 269) spacers were observed at all three time points, 4.33 % (748/17 269) were shared between at birth and 4 months, 6.22 % (1074/17 269) were shared between at 4 month and 12 months (Fig. 3d). At the individual level, spacers shared at different times were scarce (Fig. 3e). This result also verified spacers probably disappeared in the microbiome over time.

In addition to the simple prediction that cCRISPRs and spacers follow the changes of bacterial abundance and new colonization of bacteria, we observed some changes that are inconsistent with bacterial changes. The Jaccard index analysis demonstrated that similarity in spacer composition between different time points was significantly lower than similarity in the bacterial species composition (Fig. 3f). To summarize, we observed turnover in the composition of cCRISPRs and spacers in gut microbiome during the first year of life.

### Spacer dynamics in one cCRISPR

**Fig. 4.**
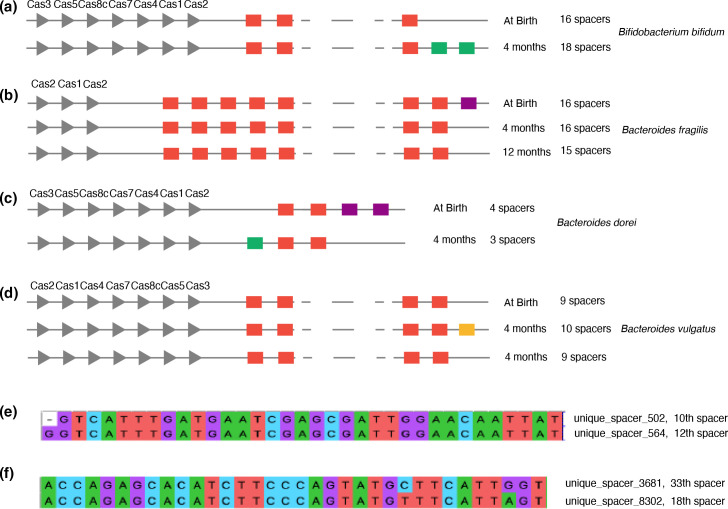
Spacer dynamics in one cCRISPR. (**a-d**) Examples of three types of changes in spacers in one cCRISPR, acquired (**a**), lost (**b**), acquired and lost simultaneously (**c**), acquired then lost (**d**). Green indicated acquired spacers, purple indicated lost spacers, yellow indicated acquired then lost spacer. (**e**) Compared with unique spacer 564, unique spacer 502 only lost the initial G, and they were the 10th and 12th spacer of one cCRISPR in baby 7 at 12 months. (**f**) Compared to unique spacer 3681, unique spacer 8302 changed C into T and G into A and they were the 33rd spacer and 18th spacer in one cCRISPR in baby 332 at birth.

To understand spacer dynamics within single cCRISPR arrays, we defined putative identical CRISPR arrays (criteria see the Methods). Overall, we detected 76 putative identical cCRISPR arrays from 46 infants (these arrays were shown in Additional file 8). Of these, 60 putative identical cCRISPR arrays were recovered at two time points, 16 were at three time points. Among them, spacers in 31 arrays (40.49 %) changed. In the changed cCRISPRs, three cases were observed: 17 arrays acquired new spacers (Fig. 4a), eight arrays lost old spacers (Fig. 4b), and six arrays both acquired new spacers and lost old spacers (Fig. 4c). More interestingly, among arrays that were recovered from three time points, we observed spacers that were longitudinally acquired then lost or lost then acquired (Fig. 4d). In most cases, one or two spacers were acquired or lost from a distant location in the CRISPR cassettes. We studied if differing bacteria taxonomy (Table 1) or differing Cas types (Table 2) showed a particular pattern of acquisition and loss, but no clear pattern was observed.

**Table 1.** Summary of spacer acquisition and loss in bacterial species

Bacteria	Acquire	Loss	Acquire and loss
* Bacteroides fragilis *	5	1	3
* Escherichia coli *	6	1	0
* Bacteroides dorei *	2	1	1
* Parabacteroides distasonis *	1	1	1
* Eubacterium rectale *	1	1	0
*Bacteroides sp.A1C1*	0	1	0
* Bacteroides vulgatus *	0	0	1
* Bifidobacterium longum *	1	0	0
* Bifidobacterium pseudocatenulatum *	1	0	0
* Enterococcus faecalis *	0	1	0
* Megamonas hypermegale *	0	1	0

**Table 2.** Summary of spacer acquisition and loss in Cas Types

Cas type	Acquire	Loss	Acquire and loss
Cas-TypeIIC	4	3	0
CAS-TypeIIIB	3	0	2
CAS-TypeIC	1	2	2
CAS-TypeIE	3	1	0
CAS-TypeIF	3	0	0
Unclassified CasType	1	0	1
CAS-TypeIU	1	0	0
CAS-TypeVIA	1	0	0
CAS-TypeIIIC	0	0	1
CAS-TypeIIA	0	1	0
CAS-TypeIIIA	0	1	0

We searched for possible instances of mutation occurring within one single cCRISPR that was recovered at multiple time points, but none was observed. Our analyses did reveal, however, 27 cases of spacer pairs within a single cCRISPR array exhibiting high similarity of nucleotides. Among these pairs, more than half of the cases differed at one single base at either direction (Fig. 4e). Notably, we also identified nine instances of single nucleotide mutation distinguishing two different spacers in one single cCRISPR array (Fig. 4f, these spacers are shown in Additional file 9).

### The indicated network of bacteria-phage interaction

As spacers are used to target invading phages, they can be used to reconstruct the interaction network between bacteria and phages. First, phages with matches to our spacer dataset were identified using BLASTn of spacer sequences against an uncultured EarlyVir database [30], which contains phages in five viral classes, split into 17 viral orders, and 246 named viral families. As a result, 32.08 % (5540/17 269) spacers had a hit (matches between phage at family level and spacers were shown Additional file 11).

**Fig. 5.**
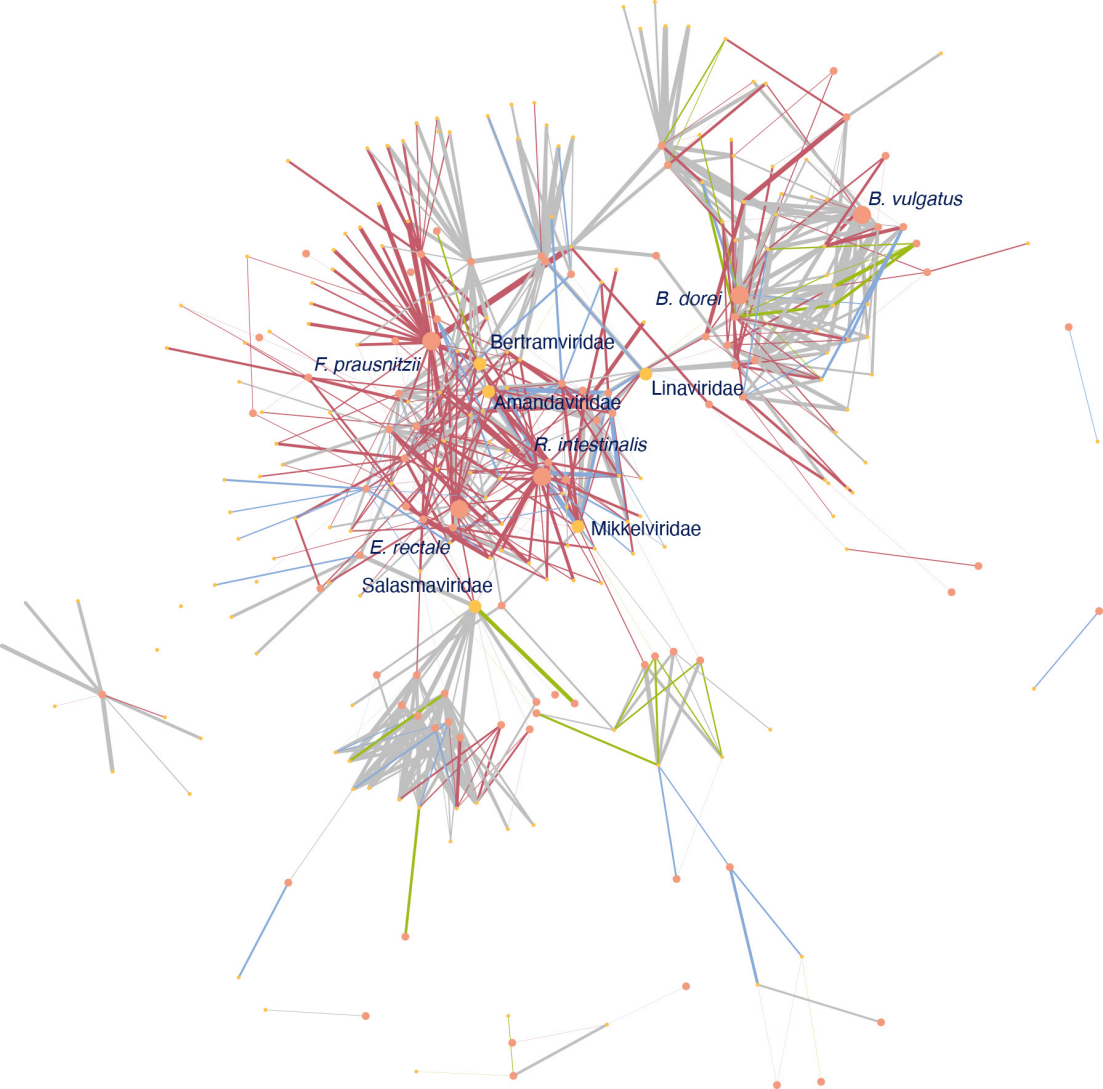
The network of bacteria-phage CRISPR defence at both bacterial species and phage family level. Grey indicates shared interactions at different time points. Green indicates interaction only reconstructed at birth, blue only at 4 months, red only at 12 months. Dark orange nodes indicate bacteria; gold nodes indicate phages.

**Fig. 6.**
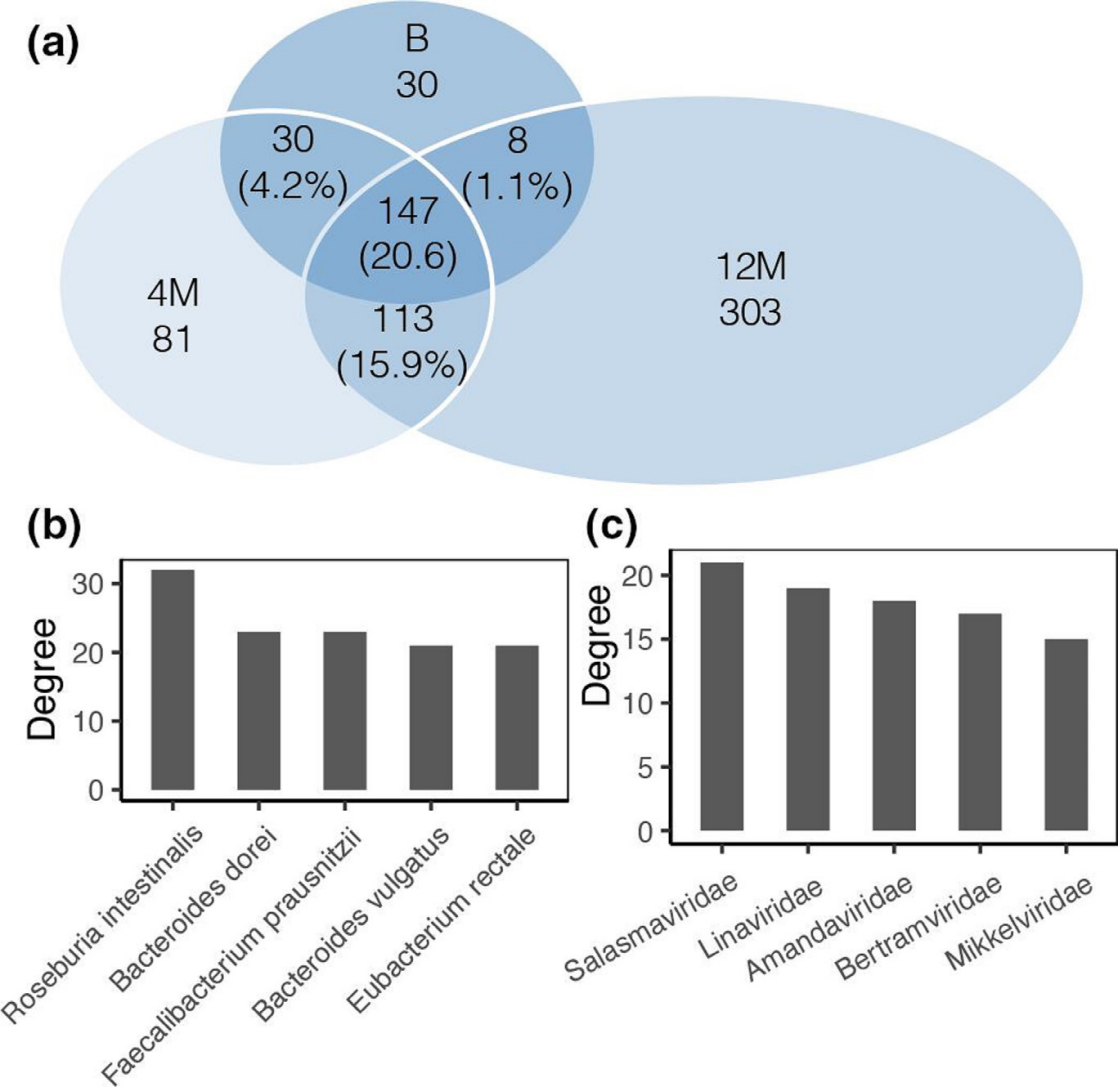
Summary of the bacteria-phage CRISPR defence network. (**a**) Venn diagram showing that most of the interactions are unique at different time points. Bacteria (**b**) and phages (**c**) with the highest degree are shown.

Then, we built the indicated network of bacteria-phage interaction (Fig. 5). In total, 712 interactions were identified between 175 named phage families and 89 bacterial species. Most of them were unique at different time points (Fig. 6a). It showed the pattern that one bacterial species interacted with multiple phages. Among bacteria, *Roseburia intestinalis, Bacteroides dorei, Faecalibacterium prausnitzii, Bacteroides vulgatus* and *Eubacterium rectale* had the most interactions (Fig. 6b). Among phages, Adamviridae, Salasmaviridae, Selmaviridae, Kasperviridae, and Arnviridae had the most interactions (Fig. 6c).

## Discussion

Our results found that CRISPR were widely distributed in gut microbiome, especially in genus *Bacteroides* and *Bifidobacterium *, consistent with a previous study [34]. We also demonstrated that the number of spacers increased with bacterial richness, which was not surprising because 1) in early life, dramatic increases in bacterial abundance lead the number of CRISPRs and spacers to change along with it; 2) as babies grow up, colonization of new bacteria brings new CRISPRs and spacers. Beyond these two processes, we also found that spacers underwent large-scale turnover in the whole gut microbiome, that is, they were recovered at one time point but not at another. Accordingly, the indicated network of bacteria-phage interaction was distinct at different times, consistent with highly dynamic interactions between bacteria and phages in the gut microbiome in early life.

In putative identical CRISPR arrays, we found richness increase and loss of spacers longitudinally. More specifically, CRISPRs incorporated new spacers into the arrays to provide defence against phages while losing other spacers, which was concordant with the stable number of spacers in one CRISPR. This could reflect the balance between protecting against phages and the cost of auto-immunity that targets their own genomes [35]. Further, we discovered single nucleotide mutation between spacers in one CRISPR, which could have resulted from acquisition of two similar spacers simultaneously, sequential acquisition, or perhaps by a duplication event. It is possible these changes occurred by colonization of closely related bacteria with different CRISPR arrays, but they could also reflect evolution of these bacteria within infant microbiome over time. This diversity of similar spacers might help to cope with the diversity and rapid evolution of phage genomes [36].

The inconsistency between the overlap of spacers and species might be because the relatively low resolution of species-resolved analysis masked more precise elucidation. Lou *et al.,* reported that throughout the first year of life, at strain-level analysis, approximately 11 % of bacterial strains persisted [37], which is more comparable with the Jaccard index of spacers. Therefore, our low Jaccard index of spacers might reflect the transient colonization of bacterial strains or rapid strain replacement.

This study investigated the dynamics of CRISPRs in the gut microbiome during early life and indicated the dynamic interaction between bacteria and phages. Nevertheless, the limitations of short-read, shotgun sequencing followed by an assembly-based approach, particularly with respect to CRISPR repeat sequences, pose significant challenges. The human gut tract constitutes a complex and dynamic system characterized by significant physical peristalsis, whereby the presence of distinct dietary patterns on a daily basis leads to fluctuations in the growth and decay of microbiome, as well as the corresponding phages that target them. The spacer dynamics we infer here could be validated further by focusing on a single CRISPR, longer-read sequencing or culture-based enrichment of specific bacterial taxa. Additionally, the molecular and ecological mechanisms underlying the acquisition or loss of CRISPR spacers remain unknown. To shed light on these dynamics, future research should utilize deep sequencing with long read metagenomics of both the gut microbiome and virome, followed by constructing high-resolution CRISPR arrays longitudinally to monitor dynamics and interactions between bacteria and phages. This approach could elucidate the observed dynamics in greater detail and reveal whether they have any implications for the host’s physiology and disease status. Additionally, examining CRISPR dynamics in maternal gut microbiomes would facilitate the inference of vertical transmission of CRISPR-mediated phage defenses to infant, and the accumulation of CRISPR spacers over time, given the relatively greater microbial stability observed in adult microbiota.

Our results highlight the rapid changes in bacteria and phage populations, and a key trait that mediates their interaction, during this key stage in human development. Several researchers have proposed phage therapy as a method to manipulate gut microbiomes [38, 39] for instance to reduce pathogen populations or stimulate beneficial microbes. To date, however, attempts to put this into practice have met with mixed success [40]. Our results provide one possible explanation for low success rates, namely that bacterial populations already experience major changes in phage populations and are adapted to respond rapidly to them. Further investigation of CRISPRs as both a causal trait and a record of bacteria-phage interactions will help to elucidate the potential for phage therapy interventions further.

## Supplementary Data

Supplementary material 1

Supplementary material 2
