# Supplementary material for: CRISPR dynamics during the interaction between bacteria and phage in the first year of life
Source: Microb Genom. 2023 Jul 4;9(7):mgen001053. doi: 10.1099/mgen.0.001053 (PMC10438810; doi:10.1099/mgen.0.001053)
Supplement: Supplementary material 1 [file mgen-9-1053-s001.pdf]

Fig S1 | The summary of the workflow.

a

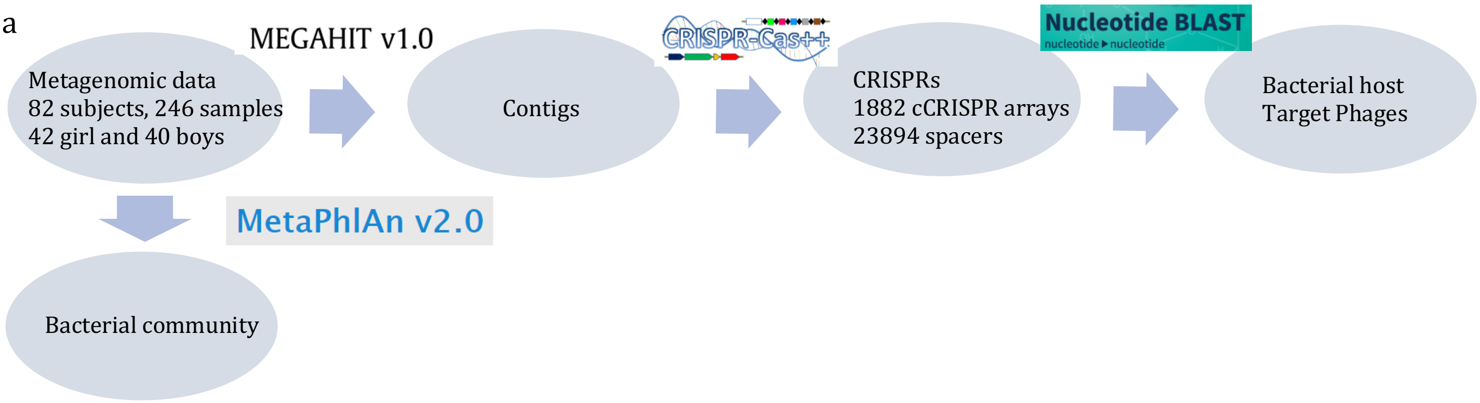

b

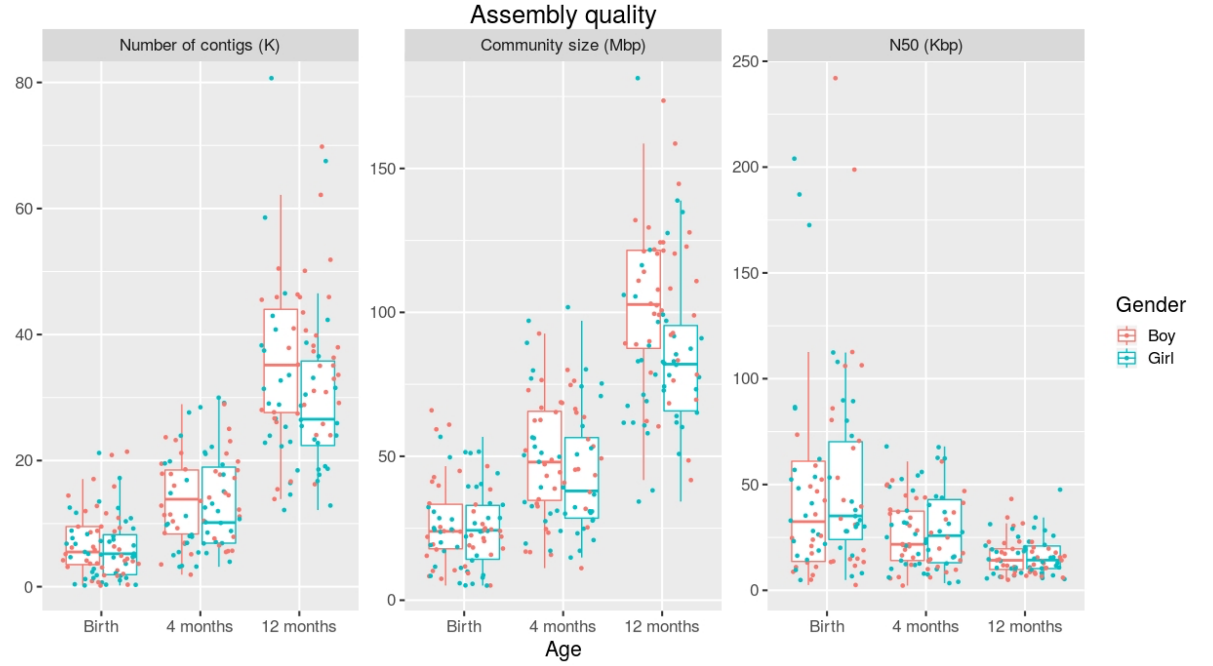

(a) Clean metagenomic data from 246 samples were assembled into contigs using MEGAHIT. Then CRISPRCasFinder was applied to identify CRISPR arrays. Bacterial hosts and target phages by spacers were assigned by BLASTn. Meanwhile, the bacterial communities were investigated by MetaPhlAn2. (b) The quality of assemblies. (a) The number of contigs (K), (b) the community size (Mbp) and (c) the N50 (Kbp) in one sample. The difference in community size between girls and boys at 12 months was significant (Mann-Whitney U test, W = 1195, P = 0.00891).

Fig S2 | Bacterial expansion with age

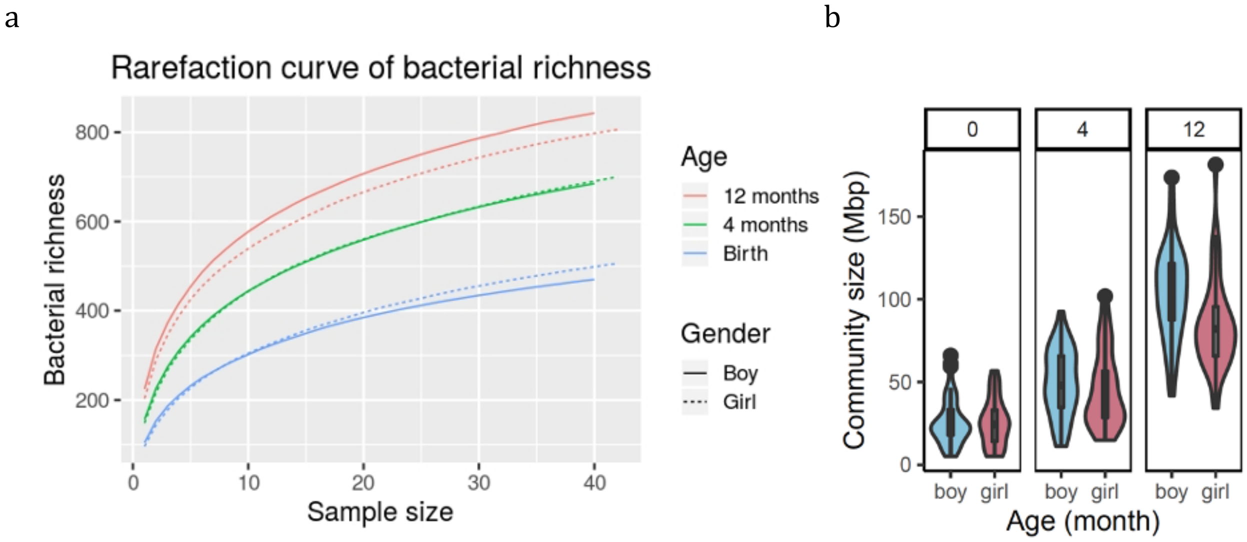

(a) The rarefaction curve of bacterial richness.
(b) Bacterial community size increased with age
